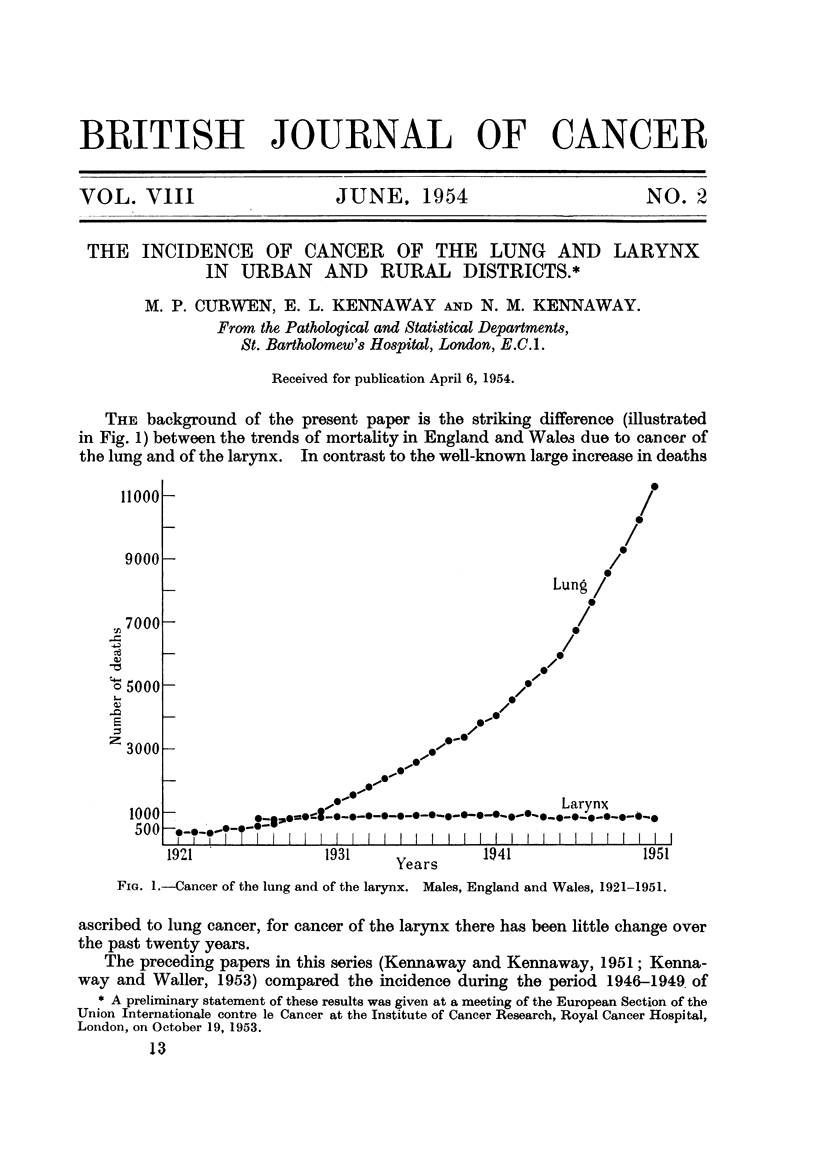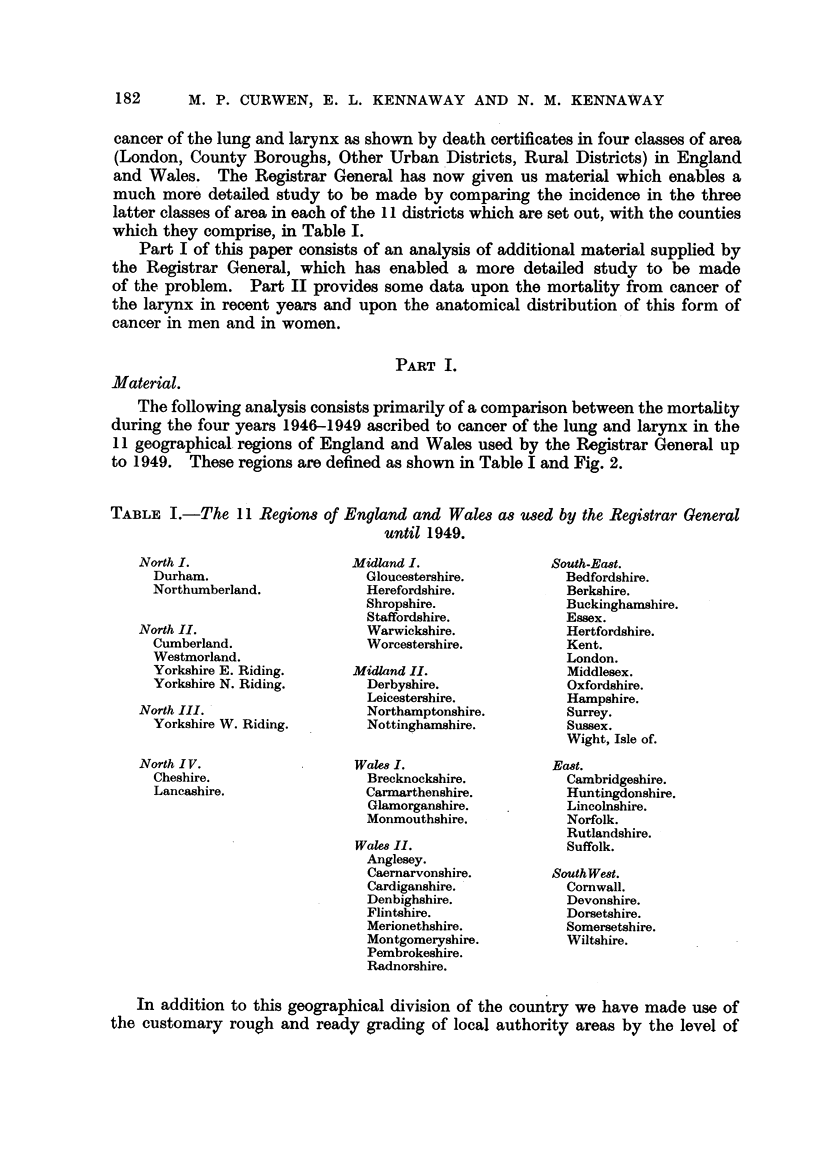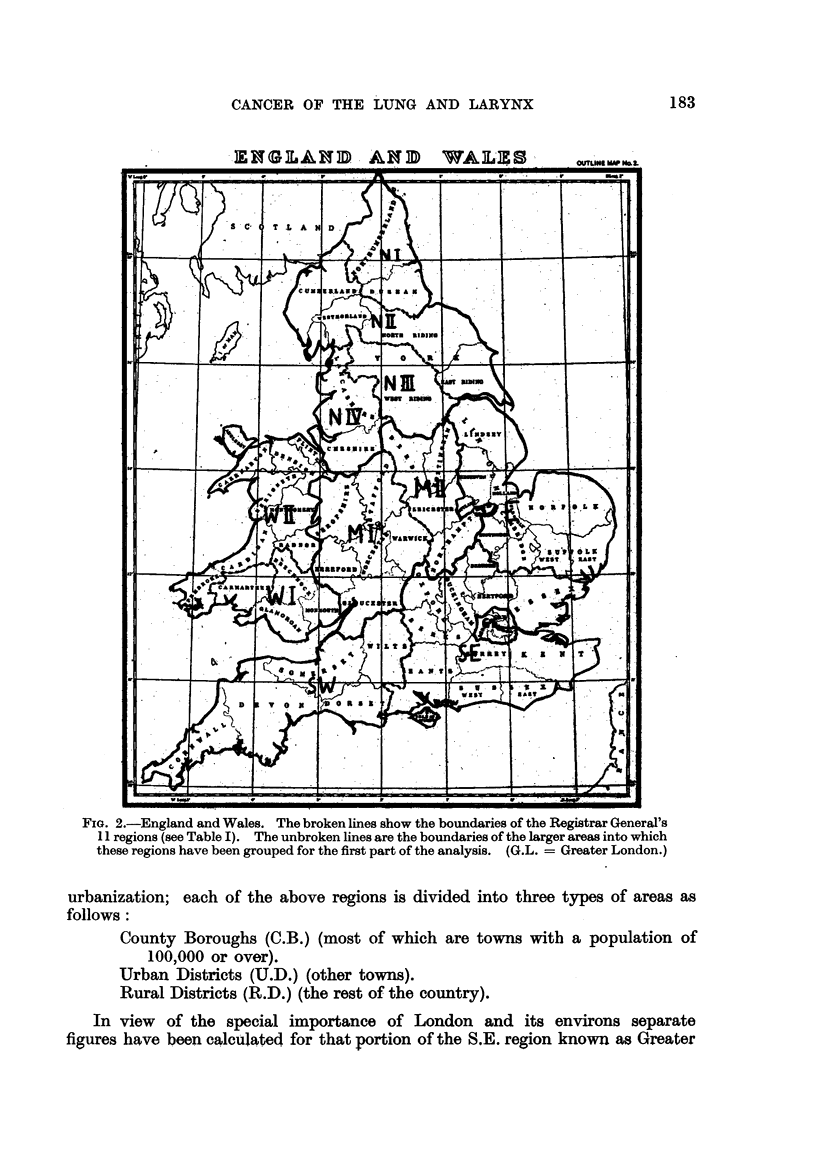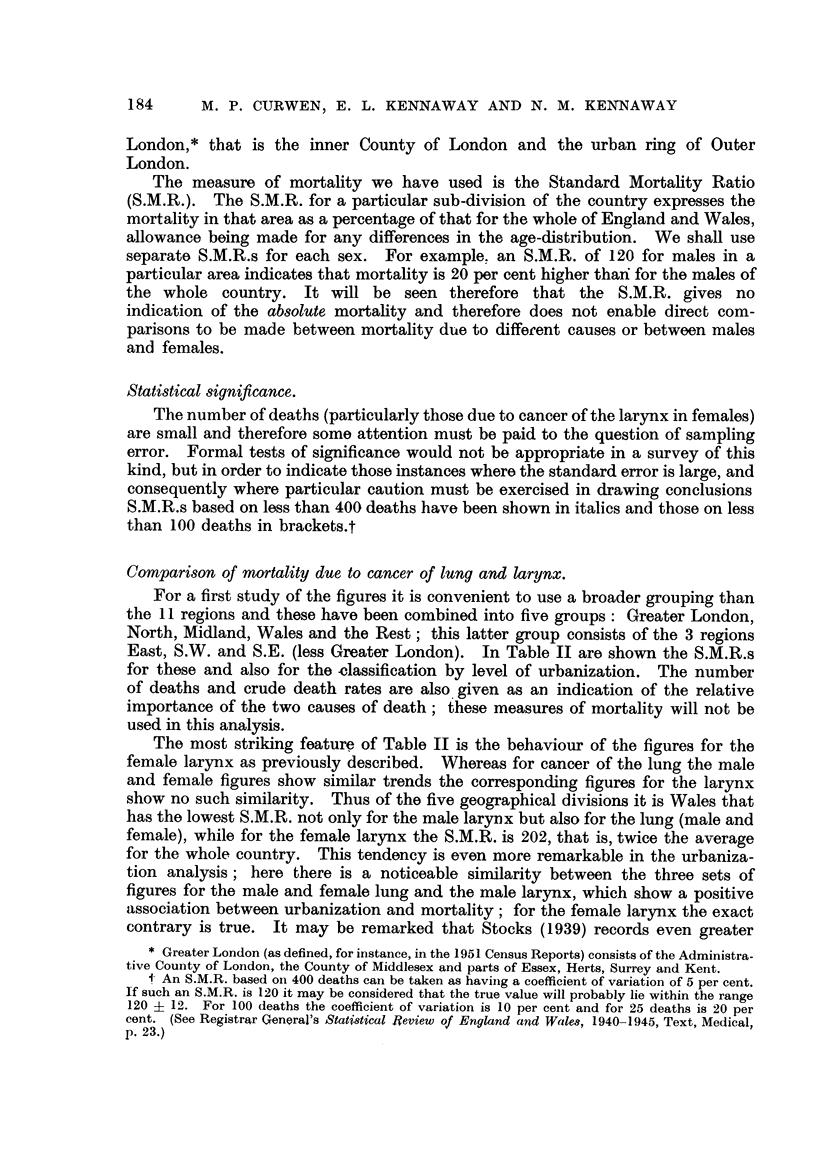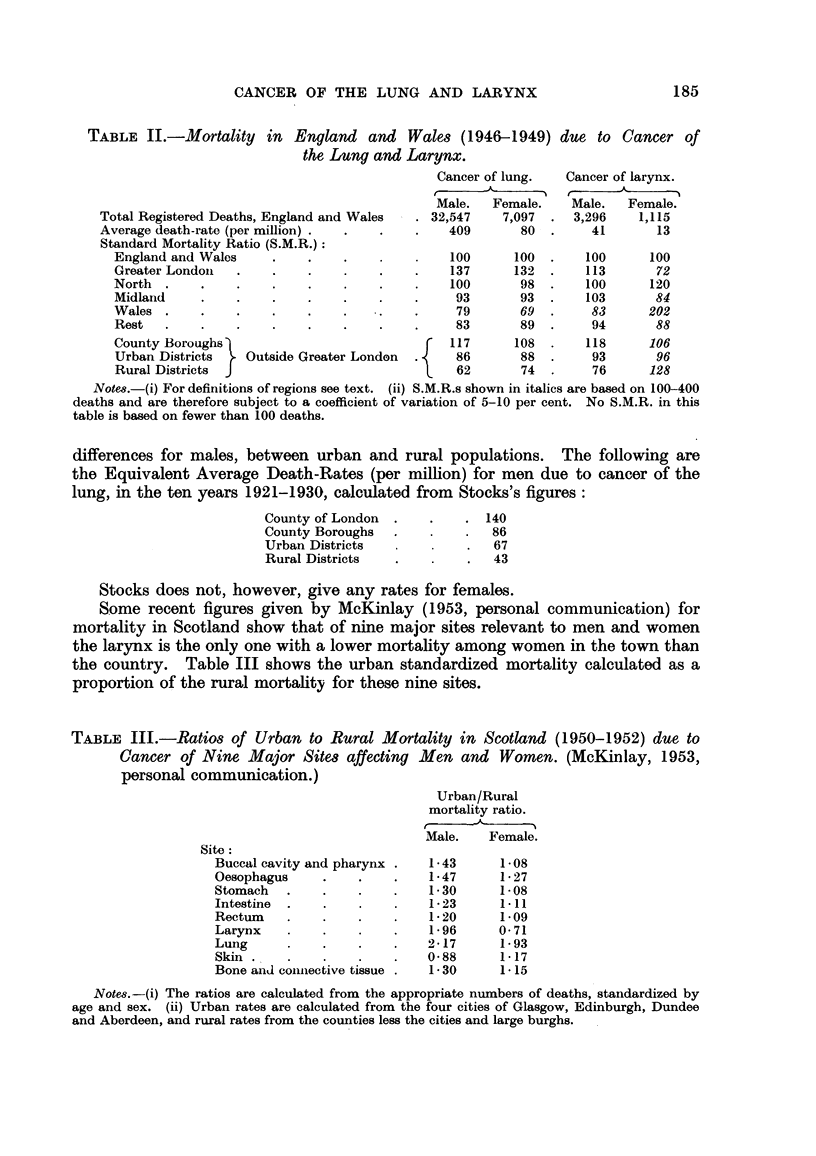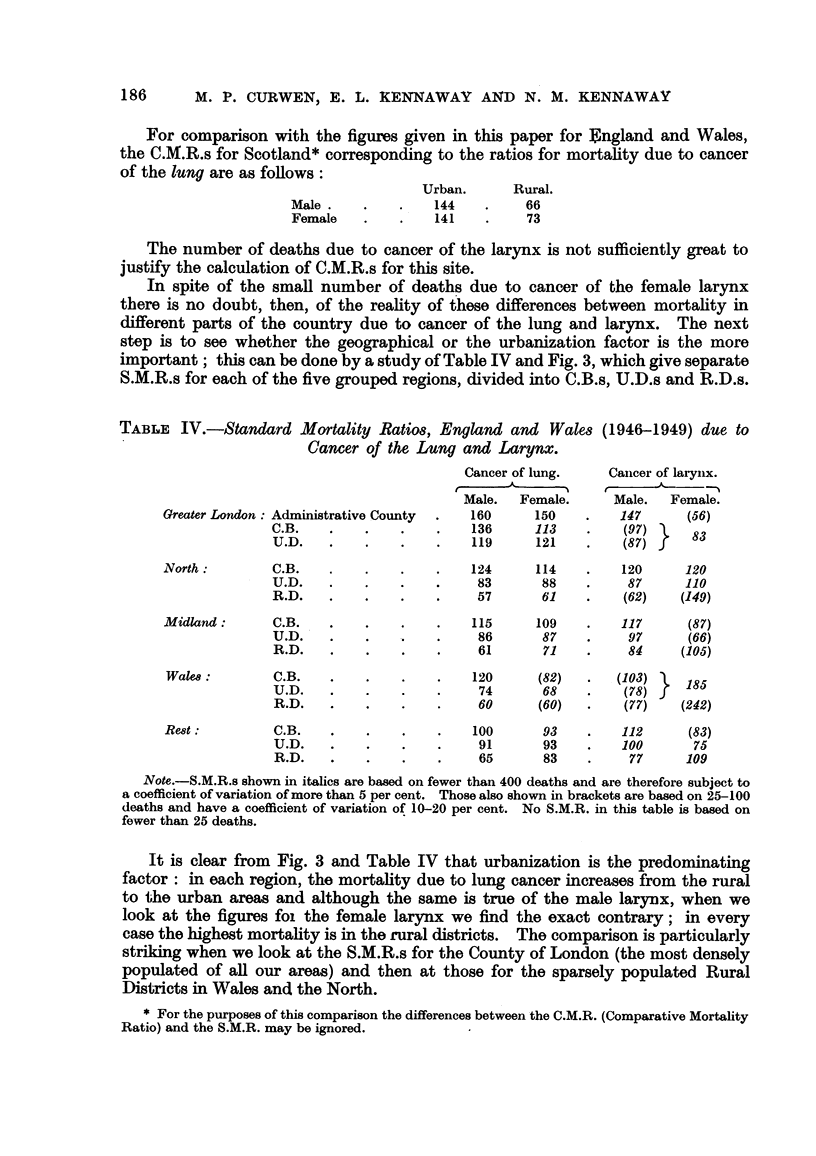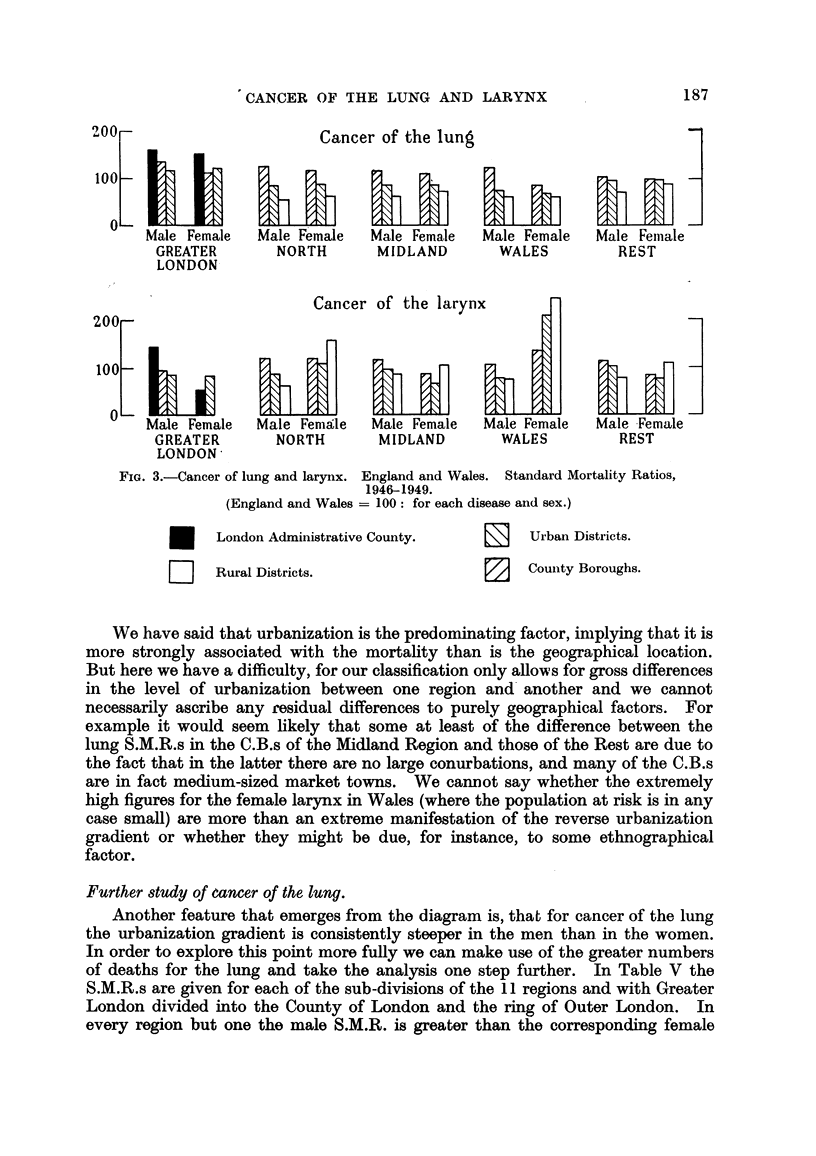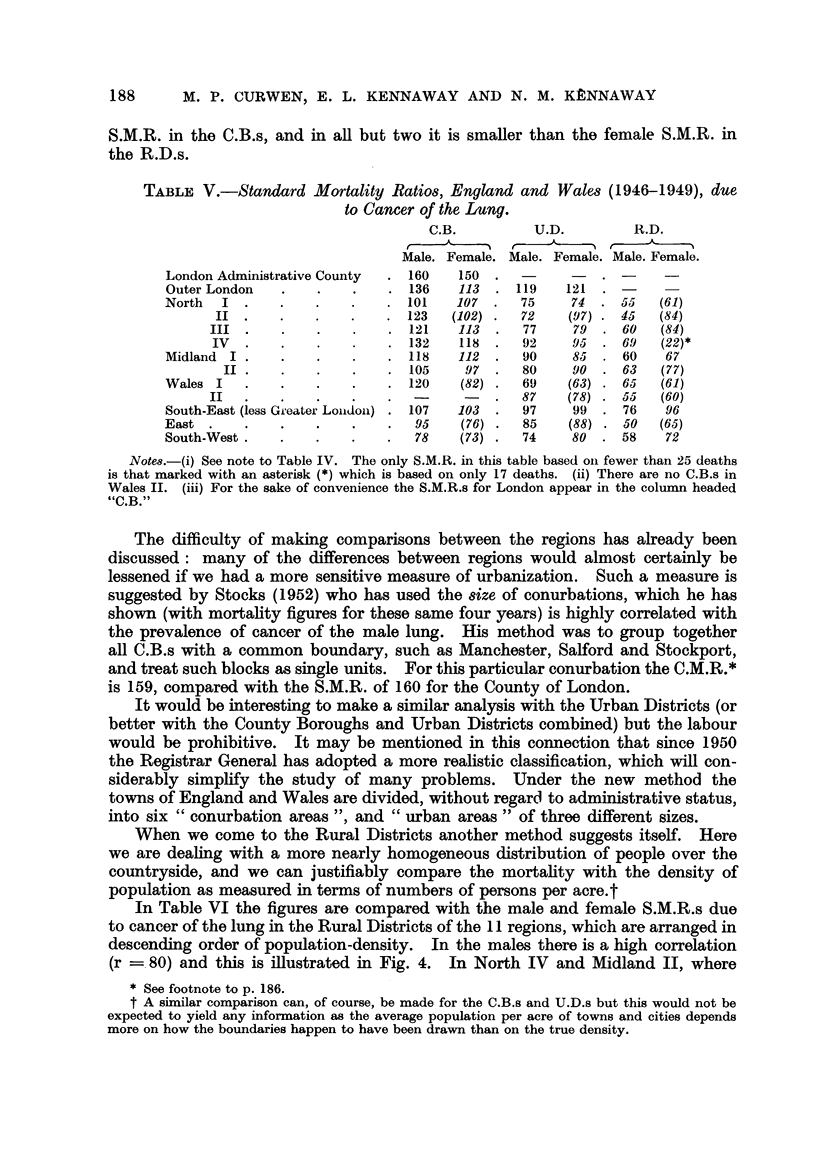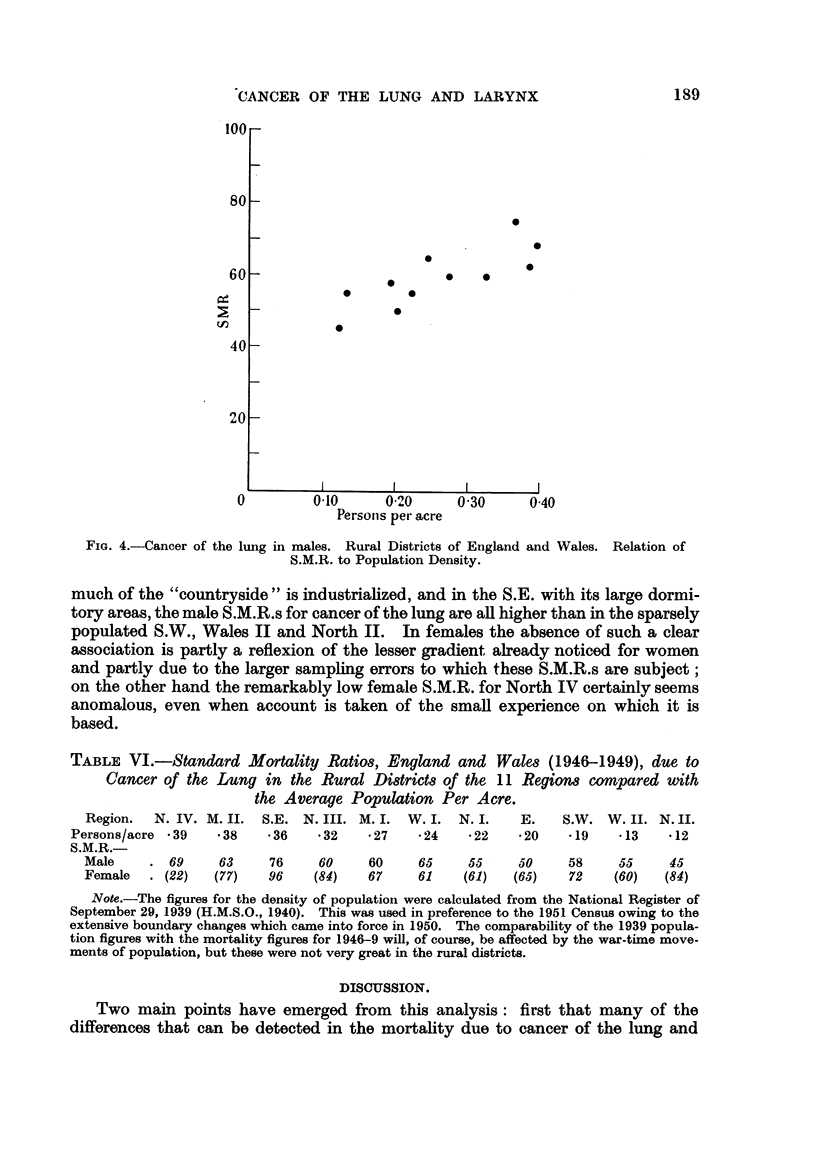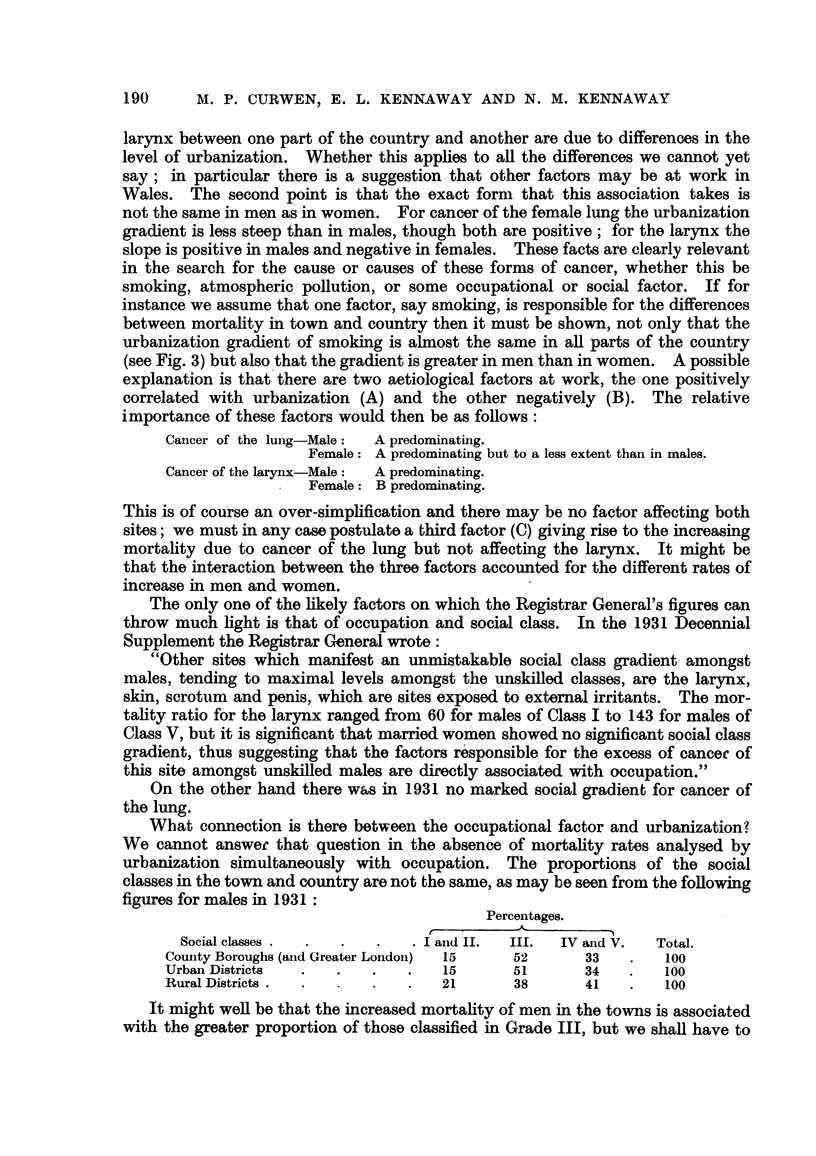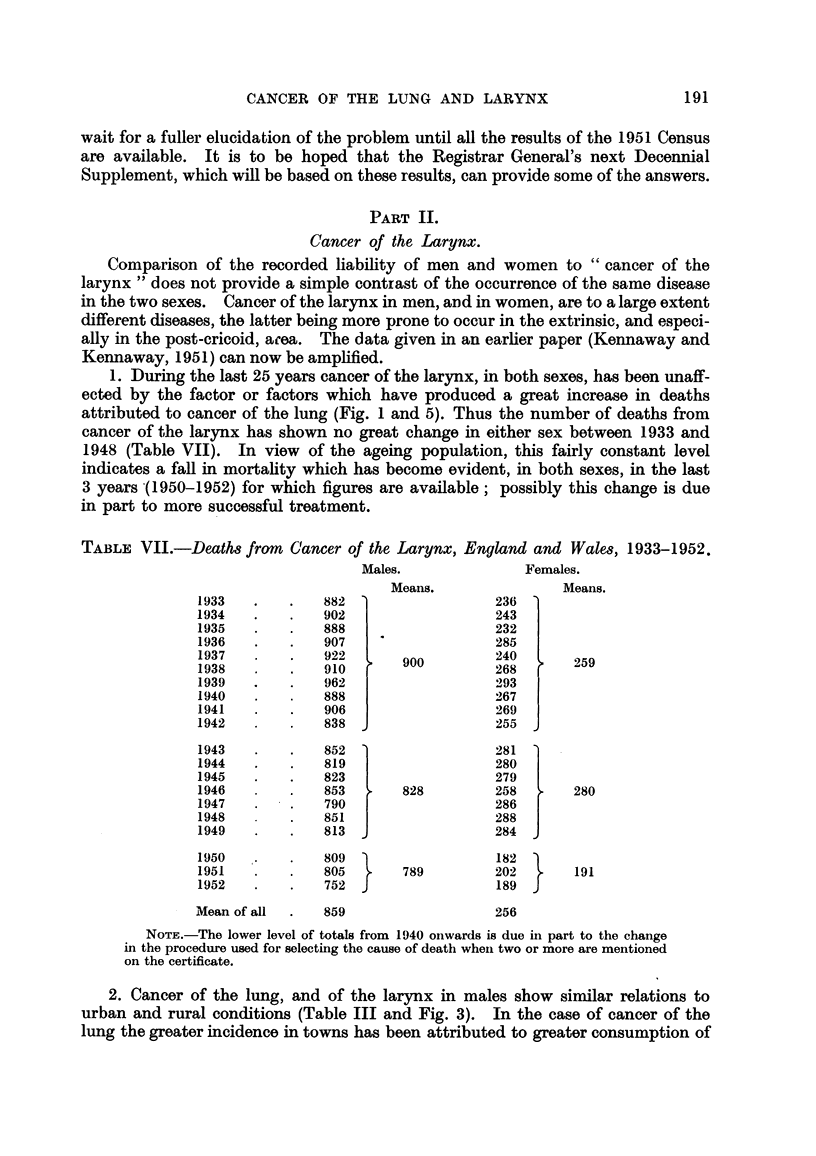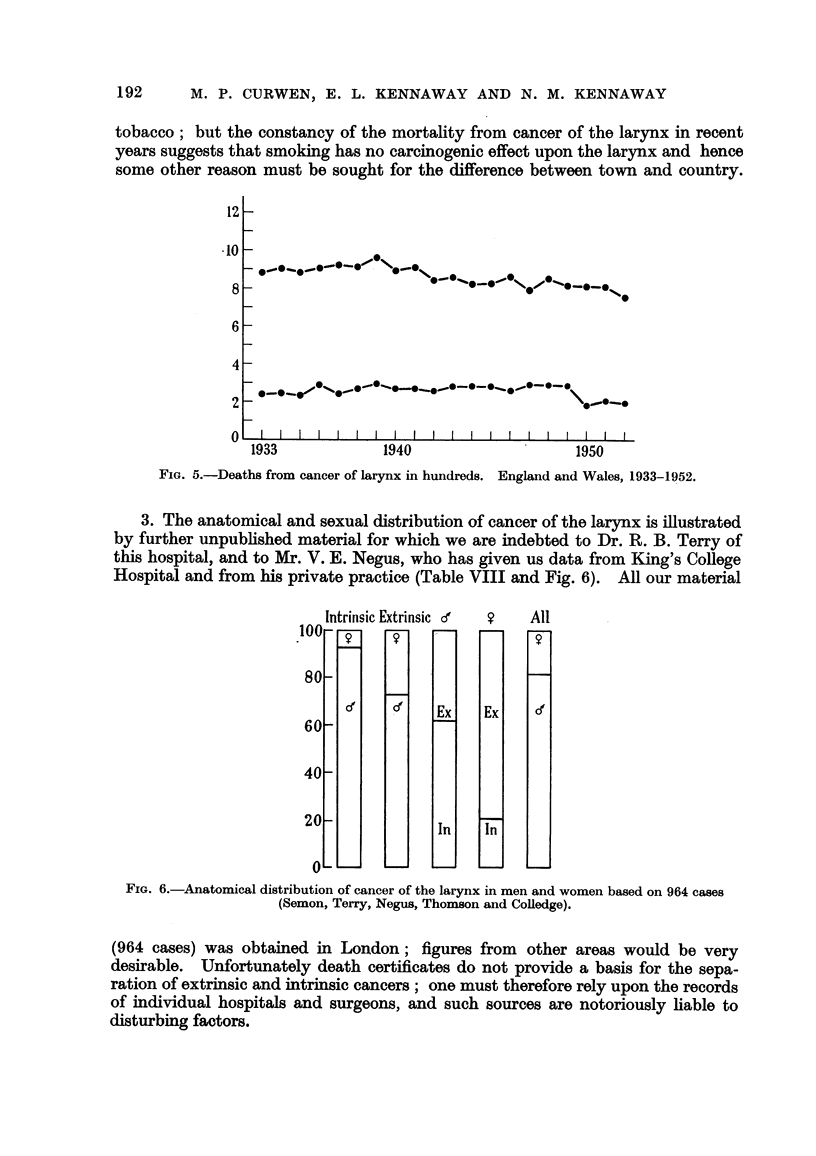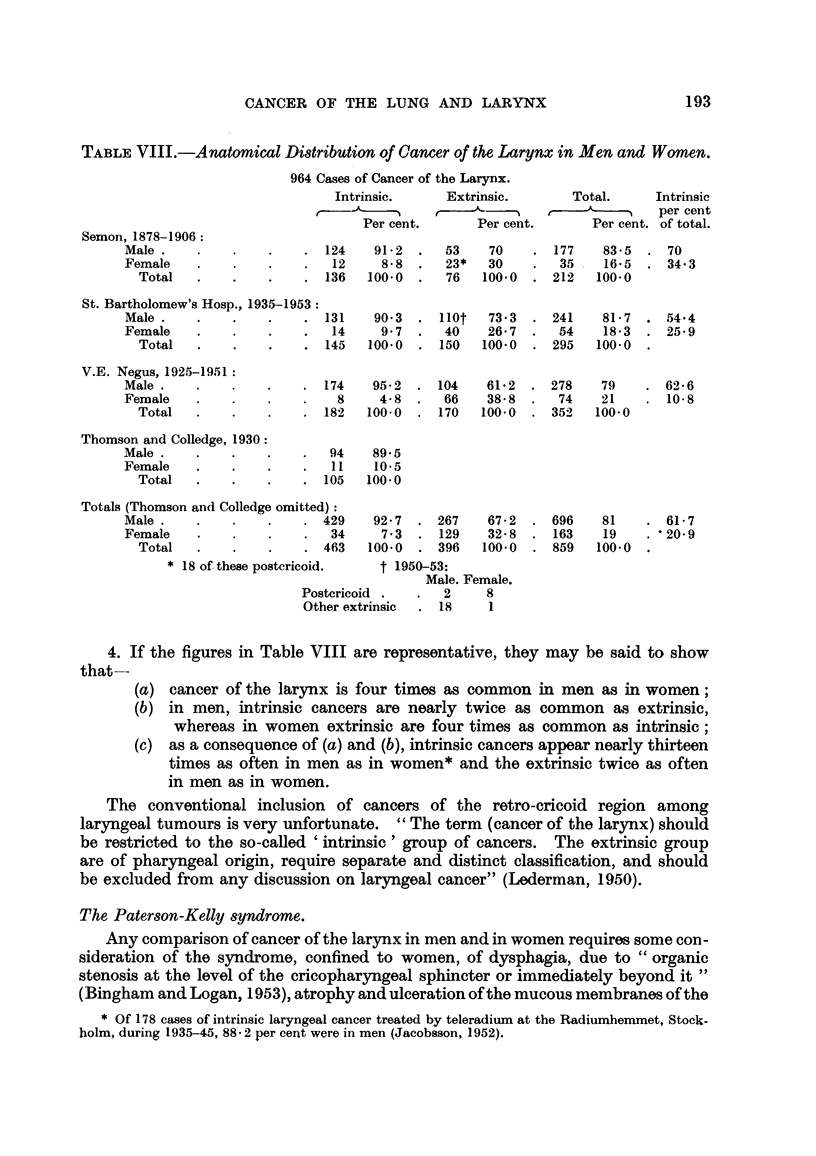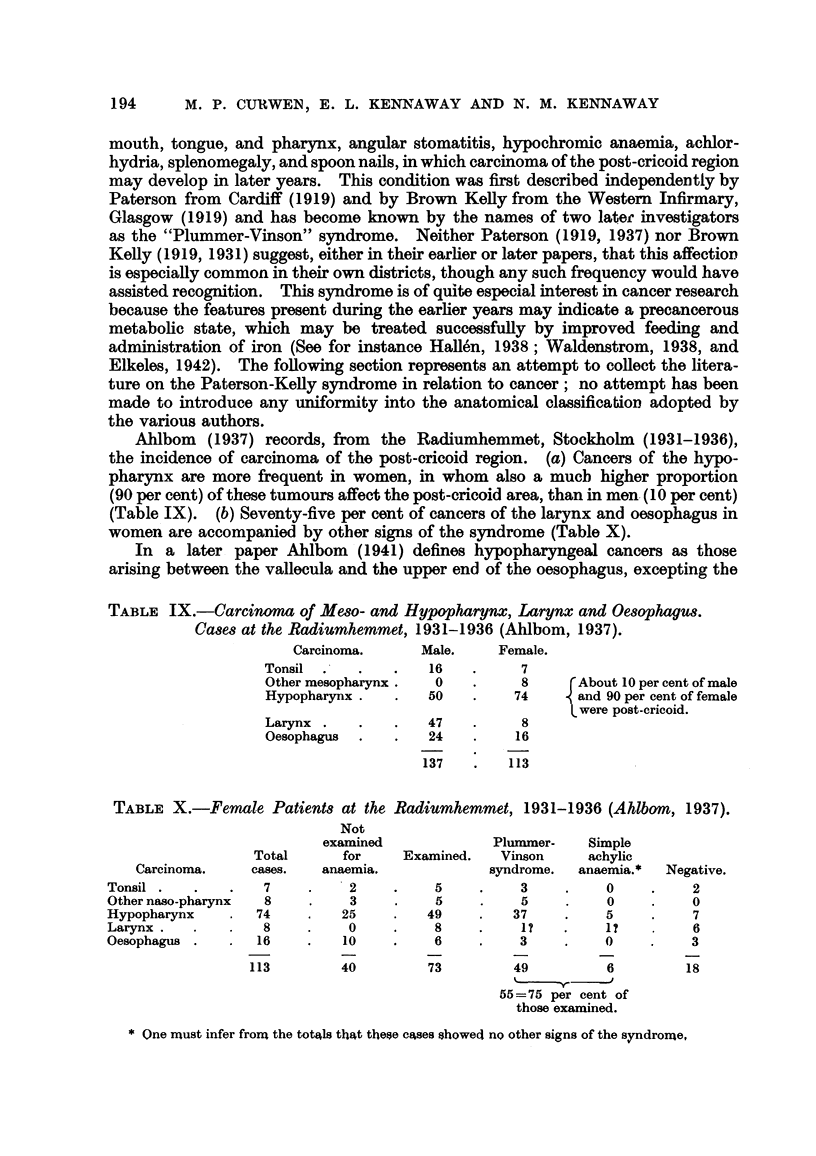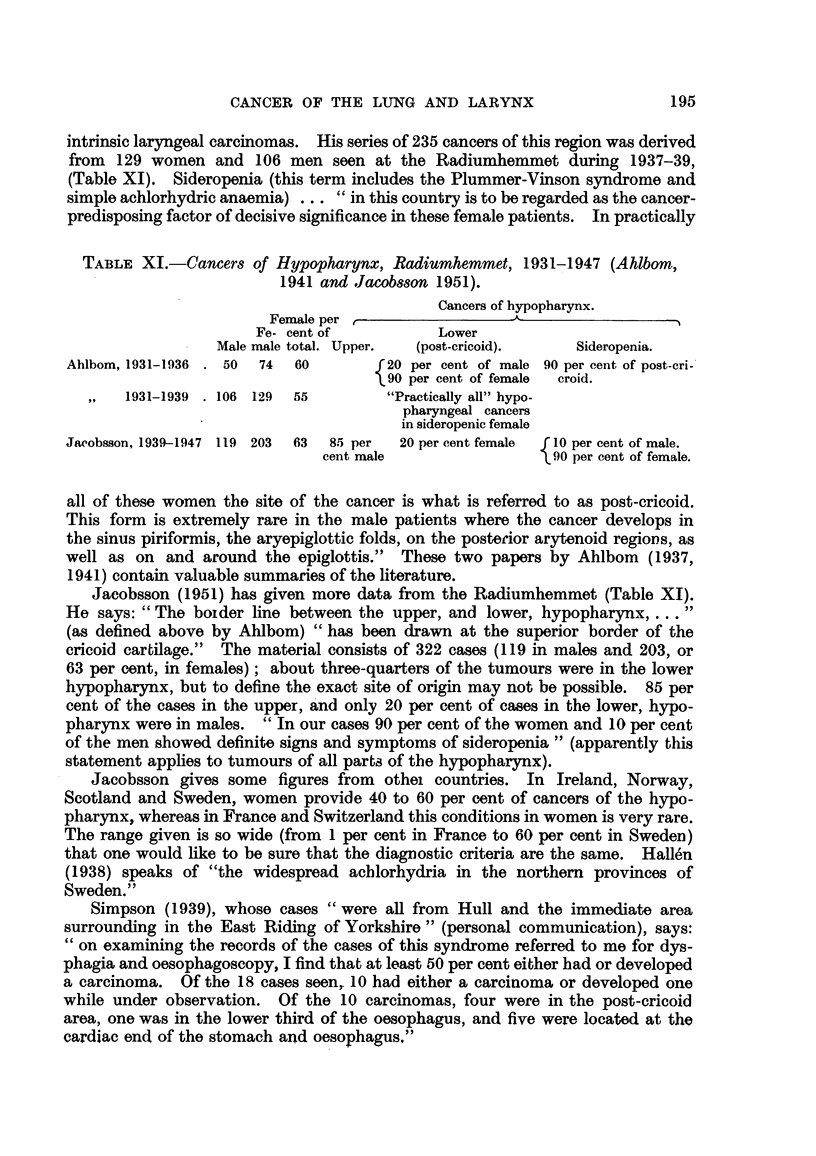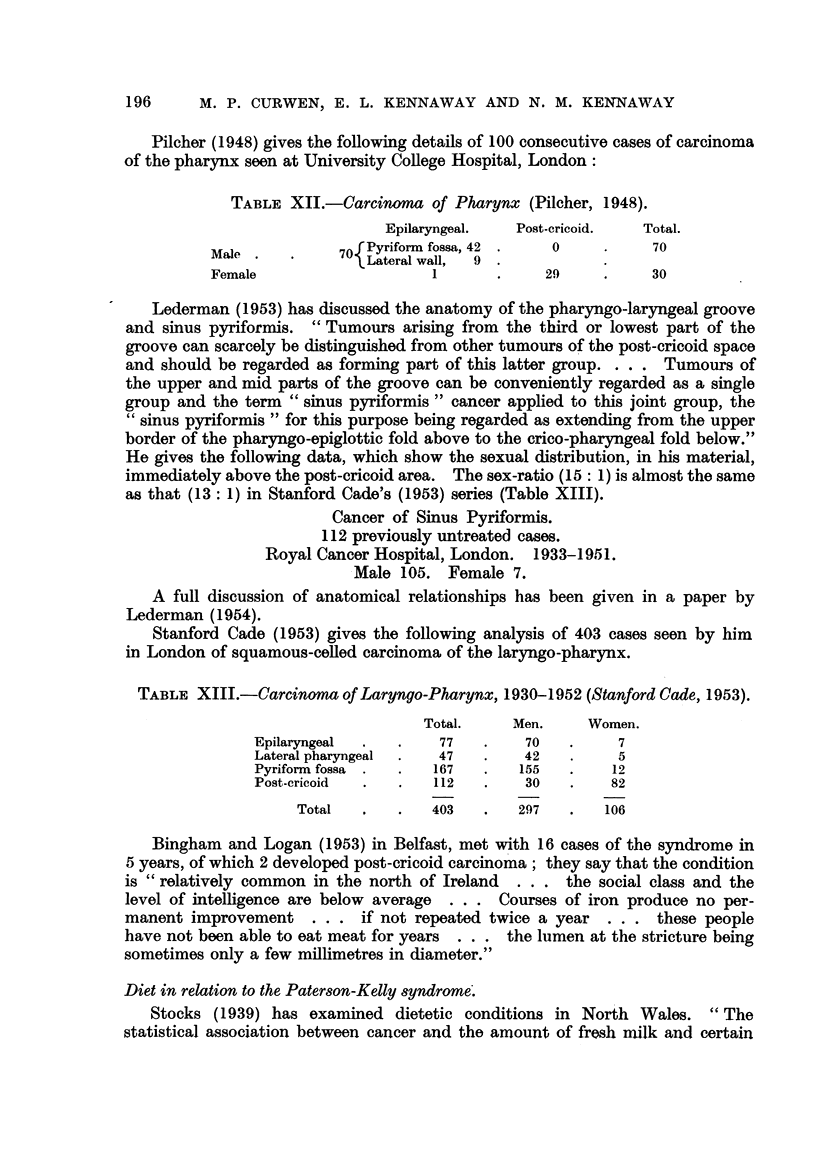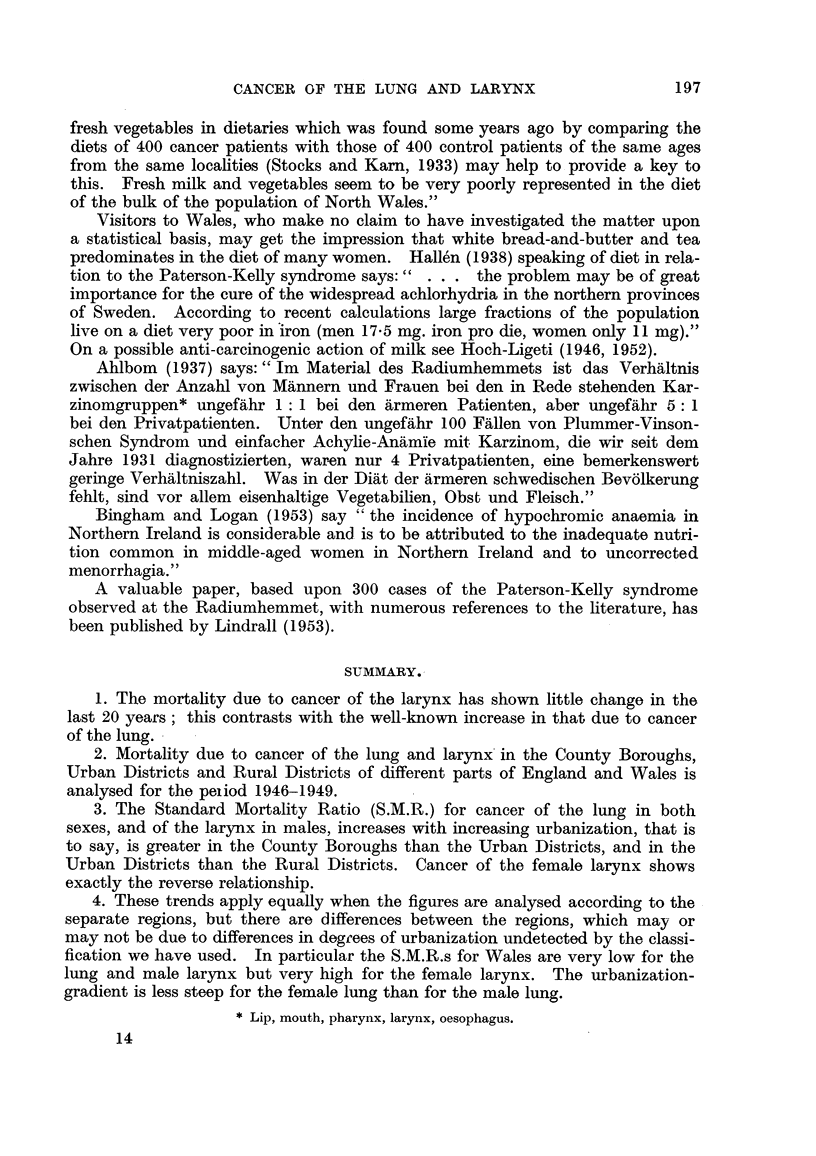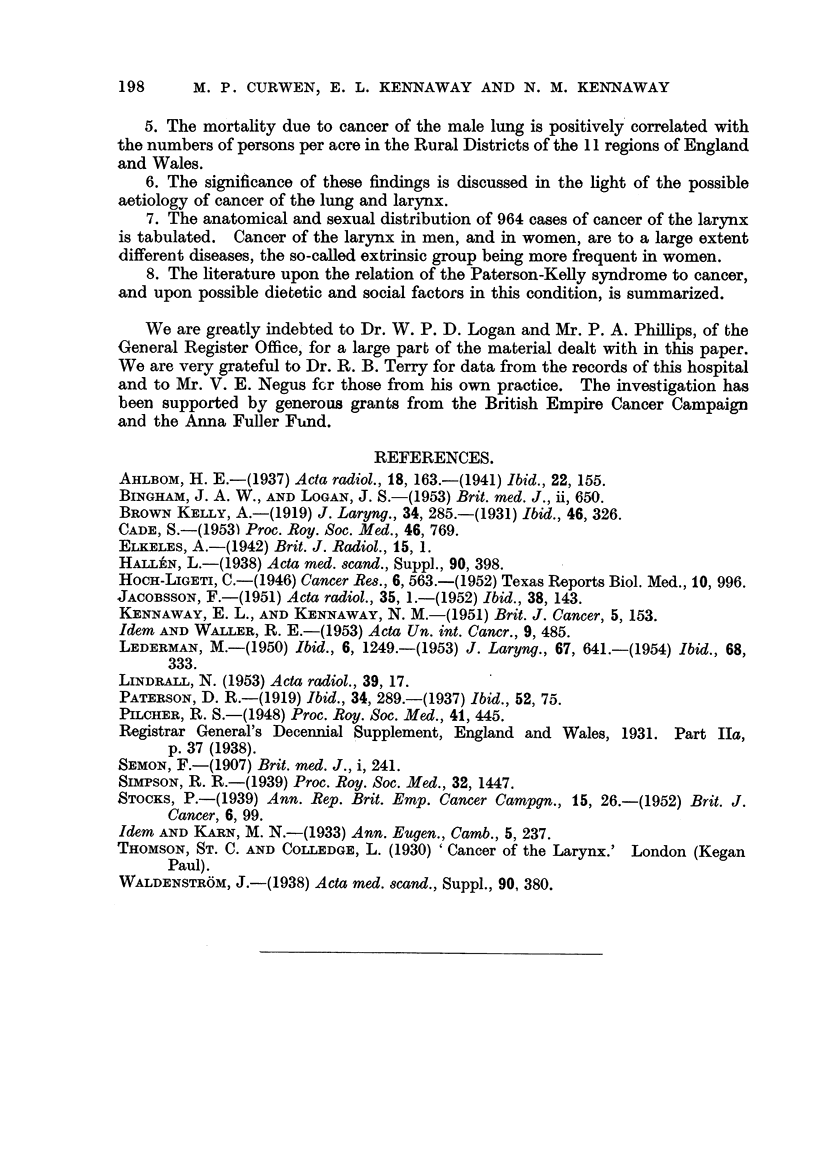# The Incidence of Cancer of the Lung and Larynx in Urban and Rural Districts*

**DOI:** 10.1038/bjc.1954.18

**Published:** 1954-06

**Authors:** M. P. Curwen, E. L. Kennaway, N. M. Kennaway


VOL. VIII JUNE, 1954 NO. 2

### THE INCIDENCE OF CANCER OF THE LUNG AND LARYNX IN URBAN AND RURAL DISTRICTS.*

M. P. CURWEN, E. L. KENNAWAY AND N. M. KENNAWAY.

*From the Pathological and Statistical Departments, St. Bartholomew's Hospital, London, E.C.1.*

Received for publication April 6, 1954.

THE background of the present paper is the striking difference (illustrated in Fig. 1) between the trends of mortality in England and Wales due to cancer of the lung and of the larynx. In contrast to the well-known large increase in deaths

FIG. 1.—Cancer of the lung and of the larynx. Males, England and Wales, 1921–1951.

ascribed to lung cancer, for cancer of the larynx there has been little change over the past twenty years.

The preceding papers in this series (Kennaway and Kennaway, 1951; Kennaway and Waller, 1953) compared the incidence during the period 1946–1949 of

* A preliminary statement of these results was given at a meeting of the European Section of the Union Internationale contre le Cancer at the Institute of Cancer Research, Royal Cancer Hospital, London, on October 19, 1953.

cancer of the lung and larynx as shown by death certificates in four classes of area (London, County Boroughs, Other Urban Districts, Rural Districts) in England and Wales. The Registrar General has now given us material which enables a much more detailed study to be made by comparing the incidence in the three latter classes of area in each of the 11 districts which are set out, with the counties which they comprise, in Table I.

Part I of this paper consists of an analysis of additional material supplied by the Registrar General, which has enabled a more detailed study to be made of the problem. Part II provides some data upon the mortality from cancer of the larynx in recent years and upon the anatomical distribution of this form of cancer in men and in women.

### Part I.

*Material.*

The following analysis consists primarily of a comparison between the mortality during the four years 1946–1949 ascribed to cancer of the lung and larynx in the 11 geographical regions of England and Wales used by the Registrar General up to 1949. These regions are defined as shown in Table I and Fig. 2.

Table I.—*The* 11 *Regions of England and Wales as used by the Registrar General until* 1949.

*North I.*

Durham.

Northumberland.

*North II.*
Cumberland.
Westmorland.
Yorkshire E. Riding.
Yorkshire N. Riding.

*North III.*
Yorkshire W. Riding.

*North IV.*
Cheshire.
Lancashire.

*Midland I.*
Gloucestershire.
Herefordshire.
Shropshire.
Staffordshire.
Warwickshire.
Worcestershire.

*Midland II.*

Derbyshire.

Leicestershire.

Northamptonshire.

Nottinghamshire.

*Wales I.*
Brecknockshire.
Carmarthenshire.
Glamorganshire.
Monmouthshire.

*Wales II.*
Anglesey.
Caernarvonshire.
Cardiganshire.
Denbighshire.
Flintshire.
Merionethshire.
Montgomeryshire.
Pembrokeshire.
Radnorshire.

*South-East.*
Bedfordshire.
Berkshire.
Buckinghamshire.
Essex.
Hertfordshire.
Kent.
London.
Middlesex.
Oxfordshire.
Hampshire.
Surrey.
Sussex.
Wight, Isle of.

*East.*
Cambridgeshire.
Huntingdonshire.
Lincolnshire.
Norfolk.
Rutlandshire.
Suffolk.

*South West.*
Cornwall.
Devonshire.
Dorsetshire.
Somersetshire.
Wiltshire.

In addition to this geographical division of the country we have made use of the customary rough and ready grading of local authority areas by the level of

FIG. 2.—England and Wales. The broken lines show the boundaries of the Registrar General's 11 regions (see Table I). The unbroken lines are the boundaries of the larger areas into which these regions have been grouped for the first part of the analysis. (G.L. = Greater London.)

urbanization; each of the above regions is divided into three types of areas as follows :

County Boroughs (C.B.) (most of which are towns with a population of 100,000 or over).
Urban Districts (U.D.) (other towns).
Rural Districts (R.D.) (the rest of the country).

In view of the special importance of London and its environs separate figures have been calculated for that portion of the S.E. region known as Greater

London,* that is the inner County of London and the urban ring of Outer London.

The measure of mortality we have used is the Standard Mortality Ratio (S.M.R.). The S.M.R. for a particular sub-division of the country expresses the mortality in that area as a percentage of that for the whole of England and Wales, allowance being made for any differences in the age-distribution. We shall use separate S.M.R.s for each sex. For example, an S.M.R. of 120 for males in a particular area indicates that mortality is 20 per cent higher than for the males of the whole country. It will be seen therefore that the S.M.R. gives no indication of the *absolute* mortality and therefore does not enable direct comparisons to be made between mortality due to different causes or between males and females.

*Statistical significance.*

The number of deaths (particularly those due to cancer of the larynx in females) are small and therefore some attention must be paid to the question of sampling error. Formal tests of significance would not be appropriate in a survey of this kind, but in order to indicate those instances where the standard error is large, and consequently where particular caution must be exercised in drawing conclusions S.M.R.s based on less than 400 deaths have been shown in italics and those on less than 100 deaths in brackets.†

*Comparison of mortality due to cancer of lung and larynx.*

For a first study of the figures it is convenient to use a broader grouping than the 11 regions and these have been combined into five groups : Greater London, North, Midland, Wales and the Rest ; this latter group consists of the 3 regions East, S.W. and S.E. (less Greater London). In Table II are shown the S.M.R.s for these and also for the classification by level of urbanization. The number of deaths and crude death rates are also given as an indication of the relative importance of the two causes of death ; these measures of mortality will not be used in this analysis.

The most striking feature of Table II is the behaviour of the figures for the female larynx as previously described. Whereas for cancer of the lung the male and female figures show similar trends the corresponding figures for the larynx show no such similarity. Thus of the five geographical divisions it is Wales that has the lowest S.M.R. not only for the male larynx but also for the lung (male and female), while for the female larynx the S.M.R. is 202, that is, twice the average for the whole country. This tendency is even more remarkable in the urbanization analysis ; here there is a noticeable similarity between the three sets of figures for the male and female lung and the male larynx, which show a positive association between urbanization and mortality ; for the female larynx the exact contrary is true. It may be remarked that Stocks (1939) records even greater

* Greater London (as defined, for instance, in the 1951 Census Reports) consists of the Administrative County of London, the County of Middlesex and parts of Essex, Herts, Surrey and Kent.

† An S.M.R. based on 400 deaths can be taken as having a coefficient of variation of 5 per cent. If such an S.M.R. is 120 it may be considered that the true value will probably lie within the range 120 ± 12. For 100 deaths the coefficient of variation is 10 per cent and for 25 deaths is 20 per cent. (See Registrar General's *Statistical Review of England and Wales*, 1940–1945, Text, Medical, p. 23.)

TABLE II.—*Mortality in England and Wales* (1946–1949) *due to Cancer of the Lung and Larynx.*

| | Cancer of lung. | | Cancer of larynx. | |
|---|---|---|---|---|
| | Male. | Female. | Male. | Female. |
| Total Registered Deaths, England and Wales | 32,547 | 7,097 | 3,296 | 1,115 |
| Average death-rate (per million) | 409 | 80 | 41 | 13 |
| Standard Mortality Ratio (S.M.R.) : | | | | |
| England and Wales | 100 | 100 | 100 | 100 |
| Greater London | 137 | 132 | 113 | *72* |
| North | 100 | 98 | 100 | 120 |
| Midland | 93 | 93 | 103 | *84* |
| Wales | 79 | *69* | *83* | *202* |
| Rest | 83 | 89 | 94 | *88* |
| County Boroughs (Outside Greater London) | 117 | 108 | 118 | *106* |
| Urban Districts (Outside Greater London) | 86 | 88 | 93 | *96* |
| Rural Districts (Outside Greater London) | 62 | 74 | 76 | *128* |

*Notes.*—(i) For definitions of regions see text. (ii) S.M.R.s shown in italics are based on 100–400 deaths and are therefore subject to a coefficient of variation of 5–10 per cent. No S.M.R. in this table is based on fewer than 100 deaths.

differences for males, between urban and rural populations. The following are the Equivalent Average Death-Rates (per million) for men due to cancer of the lung, in the ten years 1921–1930, calculated from Stocks's figures :

| | |
|---|---|
| County of London | 140 |
| County Boroughs | 86 |
| Urban Districts | 67 |
| Rural Districts | 43 |

Stocks does not, however, give any rates for females.

Some recent figures given by McKinlay (1953, personal communication) for mortality in Scotland show that of nine major sites relevant to men and women the larynx is the only one with a lower mortality among women in the town than the country. Table III shows the urban standardized mortality calculated as a proportion of the rural mortality for these nine sites.

TABLE III.—*Ratios of Urban to Rural Mortality in Scotland* (1950–1952) *due to Cancer of Nine Major Sites affecting Men and Women.* (McKinlay, 1953, personal communication.)

| | Urban/Rural mortality ratio. | |
|---|---|---|
| | Male. | Female. |
| Site : | | |
| Buccal cavity and pharynx | 1·43 | 1·08 |
| Oesophagus | 1·47 | 1·27 |
| Stomach | 1·30 | 1·08 |
| Intestine | 1·23 | 1·11 |
| Rectum | 1·20 | 1·09 |
| Larynx | 1·96 | 0·71 |
| Lung | 2·17 | 1·93 |
| Skin | 0·88 | 1·17 |
| Bone and connective tissue | 1·30 | 1·15 |

*Notes.*—(i) The ratios are calculated from the appropriate numbers of deaths, standardized by age and sex. (ii) Urban rates are calculated from the four cities of Glasgow, Edinburgh, Dundee and Aberdeen, and rural rates from the counties less the cities and large burghs.

For comparison with the figures given in this paper for England and Wales, the C.M.R.s for Scotland* corresponding to the ratios for mortality due to cancer of the *lung* are as follows :

| | Urban. | Rural. |
|---|---|---|
| Male | 144 | 66 |
| Female | 141 | 73 |

The number of deaths due to cancer of the larynx is not sufficiently great to justify the calculation of C.M.R.s for this site.

In spite of the small number of deaths due to cancer of the female larynx there is no doubt, then, of the reality of these differences between mortality in different parts of the country due to cancer of the lung and larynx. The next step is to see whether the geographical or the urbanization factor is the more important ; this can be done by a study of Table IV and Fig. 3, which give separate S.M.R.s for each of the five grouped regions, divided into C.B.s, U.D.s and R.D.s.

TABLE IV.—*Standard Mortality Ratios, England and Wales* (1946–1949) *due to Cancer of the Lung and Larynx.*

| | | Cancer of lung. | | Cancer of larynx. | |
|---|---|---|---|---|---|
| | | Male. | Female. | Male. | Female. |
| *Greater London :* | Administrative County | 160 | 150 | *147* | *(56)* |
| | C.B. | 136 | *113* | *(97)* | *83* |
| | U.D. | 119 | 121 | *(87)* | |
| *North :* | C.B. | 124 | 114 | 120 | *120* |
| | U.D. | 83 | 88 | *87* | *110* |
| | R.D. | 57 | *61* | *(62)* | *(149)* |
| *Midland :* | C.B. | 115 | 109 | *117* | *(87)* |
| | U.D. | 86 | *87* | *97* | *(66)* |
| | R.D. | 61 | *71* | *84* | *(105)* |
| *Wales :* | C.B. | 120 | *(82)* | *(103)* | *185* |
| | U.D. | 74 | *68* | *(78)* | |
| | R.D. | *60* | *(60)* | *(77)* | *(242)* |
| *Rest :* | C.B. | 100 | *93* | *112* | *(83)* |
| | U.D. | 91 | 93 | *100* | *75* |
| | R.D. | 65 | 83 | *77* | *109* |

*Note.*—S.M.R.s shown in italics are based on fewer than 400 deaths and are therefore subject to a coefficient of variation of more than 5 per cent. Those also shown in brackets are based on 25–100 deaths and have a coefficient of variation of 10–20 per cent. No S.M.R. in this table is based on fewer than 25 deaths.

It is clear from Fig. 3 and Table IV that urbanization is the predominating factor : in each region, the mortality due to lung cancer increases from the rural to the urban areas and although the same is true of the male larynx, when we look at the figures foı the female larynx we find the exact contrary ; in every case the highest mortality is in the rural districts. The comparison is particularly striking when we look at the S.M.R.s for the County of London (the most densely populated of all our areas) and then at those for the sparsely populated Rural Districts in Wales and the North.

* For the purposes of this comparison the differences between the C.M.R. (Comparative Mortality Ratio) and the S.M.R. may be ignored.

FIG. 3.—Cancer of lung and larynx. England and Wales. Standard Mortality Ratios, 1946–1949.
(England and Wales = 100 : for each disease and sex.)

London Administrative County. Urban Districts.
Rural Districts. County Boroughs.

We have said that urbanization is the predominating factor, implying that it is more strongly associated with the mortality than is the geographical location. But here we have a difficulty, for our classification only allows for gross differences in the level of urbanization between one region and another and we cannot necessarily ascribe any residual differences to purely geographical factors. For example it would seem likely that some at least of the difference between the lung S.M.R.s in the C.B.s of the Midland Region and those of the Rest are due to the fact that in the latter there are no large conurbations, and many of the C.B.s are in fact medium-sized market towns. We cannot say whether the extremely high figures for the female larynx in Wales (where the population at risk is in any case small) are more than an extreme manifestation of the reverse urbanization gradient or whether they might be due, for instance, to some ethnographical factor.

*Further study of cancer of the lung.*

Another feature that emerges from the diagram is, that for cancer of the lung the urbanization gradient is consistently steeper in the men than in the women. In order to explore this point more fully we can make use of the greater numbers of deaths for the lung and take the analysis one step further. In Table V the S.M.R.s are given for each of the sub-divisions of the 11 regions and with Greater London divided into the County of London and the ring of Outer London. In every region but one the male S.M.R. is greater than the corresponding female

S.M.R. in the C.B.s, and in all but two it is smaller than the female S.M.R. in the R.D.s.

TABLE V.—*Standard Mortality Ratios, England and Wales* (1946–1949), *due to Cancer of the Lung.*

| | C.B. | | U.D. | | R.D. | |
|---|---|---|---|---|---|---|
| | Male. | Female. | Male. | Female. | Male. | Female. |
| London Administrative County | 160 | 150 | — | — | — | — |
| Outer London | 136 | *113* | 119 | 121 | — | — |
| North I | 101 | *107* | 75 | *74* | *55* | *(61)* |
| II | 123 | *(102)* | *72* | *(97)* | *45* | *(84)* |
| III | 121 | *113* | 77 | *79* | *60* | *(84)* |
| IV | 132 | 118 | 92 | *95* | *69* | *(22)** |
| Midland I | 118 | *112* | 90 | *85* | 60 | *67* |
| II | 105 | *97* | 80 | *90* | *63* | *(77)* |
| Wales I | 120 | *(82)* | 69 | *(63)* | *65* | *(61)* |
| II | — | — | *87* | *(78)* | *55* | *(60)* |
| South-East (less Greater London) | 107 | *103* | 97 | 99 | 76 | *96* |
| East | *95* | *(76)* | 85 | *(88)* | *50* | *(65)* |
| South-West | *78* | *(73)* | 74 | *80* | 58 | *72* |

*Notes.*—(i) See note to Table IV. The only S.M.R. in this table based on fewer than 25 deaths is that marked with an asterisk (*) which is based on only 17 deaths. (ii) There are no C.B.s in Wales II. (iii) For the sake of convenience the S.M.R.s for London appear in the column headed "C.B."

The difficulty of making comparisons between the regions has already been discussed: many of the differences between regions would almost certainly be lessened if we had a more sensitive measure of urbanization. Such a measure is suggested by Stocks (1952) who has used the *size* of conurbations, which he has shown (with mortality figures for these same four years) is highly correlated with the prevalence of cancer of the male lung. His method was to group together all C.B.s with a common boundary, such as Manchester, Salford and Stockport, and treat such blocks as single units. For this particular conurbation the C.M.R.* is 159, compared with the S.M.R. of 160 for the County of London.

It would be interesting to make a similar analysis with the Urban Districts (or better with the County Boroughs and Urban Districts combined) but the labour would be prohibitive. It may be mentioned in this connection that since 1950 the Registrar General has adopted a more realistic classification, which will considerably simplify the study of many problems. Under the new method the towns of England and Wales are divided, without regard to administrative status, into six " conurbation areas ", and " urban areas " of three different sizes.

When we come to the Rural Districts another method suggests itself. Here we are dealing with a more nearly homogeneous distribution of people over the countryside, and we can justifiably compare the mortality with the density of population as measured in terms of numbers of persons per acre.†

In Table VI the figures are compared with the male and female S.M.R.s due to cancer of the lung in the Rural Districts of the 11 regions, which are arranged in descending order of population-density. In the males there is a high correlation (r = .80) and this is illustrated in Fig. 4. In North IV and Midland II, where

* See footnote to p. 186.

† A similar comparison can, of course, be made for the C.B.s and U.D.s but this would not be expected to yield any information as the average population per acre of towns and cities depends more on how the boundaries happen to have been drawn than on the true density.

FIG. 4.—Cancer of the lung in males. Rural Districts of England and Wales. Relation of S.M.R. to Population Density.

much of the "countryside" is industrialized, and in the S.E. with its large dormitory areas, the male S.M.R.s for cancer of the lung are all higher than in the sparsely populated S.W., Wales II and North II. In females the absence of such a clear association is partly a reflexion of the lesser gradient already noticed for women and partly due to the larger sampling errors to which these S.M.R.s are subject; on the other hand the remarkably low female S.M.R. for North IV certainly seems anomalous, even when account is taken of the small experience on which it is based.

TABLE VI.—*Standard Mortality Ratios, England and Wales* (1946–1949), *due to Cancer of the Lung in the Rural Districts of the* 11 *Regions compared with the Average Population Per Acre.*

| Region. | N. IV. | M. II. | S.E. | N. III. | M. I. | W. I. | N. I. | E. | S.W. | W. II. | N. II. |
|---|---|---|---|---|---|---|---|---|---|---|---|
| Persons/acre | ·39 | ·38 | ·36 | ·32 | ·27 | ·24 | ·22 | ·20 | ·19 | ·13 | ·12 |
| S.M.R.— | | | | | | | | | | | |
| Male . | *69* | *63* | 76 | *60* | 60 | *65* | *55* | *50* | 58 | *55* | *45* |
| Female . | *(22)* | *(77)* | *96* | *(84)* | *67* | *61* | *(61)* | *(65)* | *72* | *(60)* | *(84)* |

*Note.*—The figures for the density of population were calculated from the National Register of September 29, 1939 (H.M.S.O., 1940). This was used in preference to the 1951 Census owing to the extensive boundary changes which came into force in 1950. The comparability of the 1939 population figures with the mortality figures for 1946–9 will, of course, be affected by the war-time movements of population, but these were not very great in the rural districts.

### DISCUSSION.

Two main points have emerged from this analysis: first that many of the differences that can be detected in the mortality due to cancer of the lung and larynx between one part of the country and another are due to differences in the level of urbanization. Whether this applies to all the differences we cannot yet say; in particular there is a suggestion that other factors may be at work in Wales. The second point is that the exact form that this association takes is not the same in men as in women. For cancer of the female lung the urbanization gradient is less steep than in males, though both are positive; for the larynx the slope is positive in males and negative in females. These facts are clearly relevant in the search for the cause or causes of these forms of cancer, whether this be smoking, atmospheric pollution, or some occupational or social factor. If for instance we assume that one factor, say smoking, is responsible for the differences between mortality in town and country then it must be shown, not only that the urbanization gradient of smoking is almost the same in all parts of the country (see Fig. 3) but also that the gradient is greater in men than in women. A possible explanation is that there are two aetiological factors at work, the one positively correlated with urbanization (A) and the other negatively (B). The relative importance of these factors would then be as follows:

| | |
|---|---|
| Cancer of the lung—Male: | A predominating. |
| Female: | A predominating but to a less extent than in males. |
| Cancer of the larynx—Male: | A predominating. |
| Female: | B predominating. |

This is of course an over-simplification and there may be no factor affecting both sites; we must in any case postulate a third factor (C) giving rise to the increasing mortality due to cancer of the lung but not affecting the larynx. It might be that the interaction between the three factors accounted for the different rates of increase in men and women.

The only one of the likely factors on which the Registrar General's figures can throw much light is that of occupation and social class. In the 1931 Decennial Supplement the Registrar General wrote:

"Other sites which manifest an unmistakable social class gradient amongst males, tending to maximal levels amongst the unskilled classes, are the larynx, skin, scrotum and penis, which are sites exposed to external irritants. The mortality ratio for the larynx ranged from 60 for males of Class I to 143 for males of Class V, but it is significant that married women showed no significant social class gradient, thus suggesting that the factors responsible for the excess of cancer of this site amongst unskilled males are directly associated with occupation."

On the other hand there was in 1931 no marked social gradient for cancer of the lung.

What connection is there between the occupational factor and urbanization? We cannot answer that question in the absence of mortality rates analysed by urbanization simultaneously with occupation. The proportions of the social classes in the town and country are not the same, as may be seen from the following figures for males in 1931:

| | Percentages. | | | |
|---|---|---|---|---|
| Social classes | I and II. | III. | IV and V. | Total. |
| County Boroughs (and Greater London) | 15 | 52 | 33 | 100 |
| Urban Districts | 15 | 51 | 34 | 100 |
| Rural Districts | 21 | 38 | 41 | 100 |

It might well be that the increased mortality of men in the towns is associated with the greater proportion of those classified in Grade III, but we shall have to wait for a fuller elucidation of the problem until all the results of the 1951 Census are available. It is to be hoped that the Registrar General's next Decennial Supplement, which will be based on these results, can provide some of the answers.

### PART II.

### *Cancer of the Larynx.*

Comparison of the recorded liability of men and women to " cancer of the larynx " does not provide a simple contrast of the occurrence of the same disease in the two sexes. Cancer of the larynx in men, and in women, are to a large extent different diseases, the latter being more prone to occur in the extrinsic, and especially in the post-cricoid, area. The data given in an earlier paper (Kennaway and Kennaway, 1951) can now be amplified.

1. During the last 25 years cancer of the larynx, in both sexes, has been unaffected by the factor or factors which have produced a great increase in deaths attributed to cancer of the lung (Fig. 1 and 5). Thus the number of deaths from cancer of the larynx has shown no great change in either sex between 1933 and 1948 (Table VII). In view of the ageing population, this fairly constant level indicates a fall in mortality which has become evident, in both sexes, in the last 3 years (1950–1952) for which figures are available ; possibly this change is due in part to more successful treatment.

TABLE VII.—*Deaths from Cancer of the Larynx, England and Wales,* 1933–1952.

| | Males. | Males. Means. | Females. | Females. Means. |
|---|---|---|---|---|
| 1933 | 882 | 900 | 236 | 259 |
| 1934 | 902 | | 243 | |
| 1935 | 888 | | 232 | |
| 1936 | 907 | | 285 | |
| 1937 | 922 | | 240 | |
| 1938 | 910 | | 268 | |
| 1939 | 962 | | 293 | |
| 1940 | 888 | | 267 | |
| 1941 | 906 | | 269 | |
| 1942 | 838 | | 255 | |
| 1943 | 852 | 828 | 281 | 280 |
| 1944 | 819 | | 280 | |
| 1945 | 823 | | 279 | |
| 1946 | 853 | | 258 | |
| 1947 | 790 | | 286 | |
| 1948 | 851 | | 288 | |
| 1949 | 813 | | 284 | |
| 1950 | 809 | 789 | 182 | 191 |
| 1951 | 805 | | 202 | |
| 1952 | 752 | | 189 | |
| Mean of all | 859 | | 256 | |

NOTE.—The lower level of totals from 1940 onwards is due in part to the change in the procedure used for selecting the cause of death when two or more are mentioned on the certificate.

2. Cancer of the lung, and of the larynx in males show similar relations to urban and rural conditions (Table III and Fig. 3). In the case of cancer of the lung the greater incidence in towns has been attributed to greater consumption of tobacco ; but the constancy of the mortality from cancer of the larynx in recent years suggests that smoking has no carcinogenic effect upon the larynx and hence some other reason must be sought for the difference between town and country.

FIG. 5.—Deaths from cancer of larynx in hundreds. England and Wales, 1933–1952.

3. The anatomical and sexual distribution of cancer of the larynx is illustrated by further unpublished material for which we are indebted to Dr. R. B. Terry of this hospital, and to Mr. V. E. Negus, who has given us data from King's College Hospital and from his private practice (Table VIII and Fig. 6). All our material

FIG. 6.—Anatomical distribution of cancer of the larynx in men and women based on 964 cases (Semon, Terry, Negus, Thomson and Colledge).

(964 cases) was obtained in London ; figures from other areas would be very desirable. Unfortunately death certificates do not provide a basis for the separation of extrinsic and intrinsic cancers ; one must therefore rely upon the records of individual hospitals and surgeons, and such sources are notoriously liable to disturbing factors.

TABLE VIII.—*Anatomical Distribution of Cancer of the Larynx in Men and Women.*

964 Cases of Cancer of the Larynx.

| | Intrinsic. | | Extrinsic. | | Total. | | Intrinsic per cent of total. |
|---|---|---|---|---|---|---|---|
| | | Per cent. | | Per cent. | | Per cent. | |
| Semon, 1878–1906 : | | | | | | | |
| Male | 124 | 91·2 | 53 | 70 | 177 | 83·5 | 70 |
| Female | 12 | 8·8 | 23* | 30 | 35 | 16·5 | 34·3 |
| Total | 136 | 100·0 | 76 | 100·0 | 212 | 100·0 | |
| St. Bartholomew's Hosp., 1935–1953 : | | | | | | | |
| Male | 131 | 90·3 | 110† | 73·3 | 241 | 81·7 | 54·4 |
| Female | 14 | 9·7 | 40 | 26·7 | 54 | 18·3 | 25·9 |
| Total | 145 | 100·0 | 150 | 100·0 | 295 | 100·0 | |
| V.E. Negus, 1925–1951 : | | | | | | | |
| Male | 174 | 95·2 | 104 | 61·2 | 278 | 79 | 62·6 |
| Female | 8 | 4·8 | 66 | 38·8 | 74 | 21 | 10·8 |
| Total | 182 | 100·0 | 170 | 100·0 | 352 | 100·0 | |
| Thomson and Colledge, 1930 : | | | | | | | |
| Male | 94 | 89·5 | | | | | |
| Female | 11 | 10·5 | | | | | |
| Total | 105 | 100·0 | | | | | |
| Totals (Thomson and Colledge omitted) : | | | | | | | |
| Male | 429 | 92·7 | 267 | 67·2 | 696 | 81 | 61·7 |
| Female | 34 | 7·3 | 129 | 32·8 | 163 | 19 | 20·9 |
| Total | 463 | 100·0 | 396 | 100·0 | 859 | 100·0 | |

* 18 of these postcricoid.

† 1950–53:

| | Male. | Female. |
|---|---|---|
| Postcricoid | 2 | 8 |
| Other extrinsic | 18 | 1 |

4. If the figures in Table VIII are representative, they may be said to show that—

(*a*) cancer of the larynx is four times as common in men as in women ;

(*b*) in men, intrinsic cancers are nearly twice as common as extrinsic, whereas in women extrinsic are four times as common as intrinsic ;

(*c*) as a consequence of (*a*) and (*b*), intrinsic cancers appear nearly thirteen times as often in men as in women* and the extrinsic twice as often in men as in women.

The conventional inclusion of cancers of the retro-cricoid region among laryngeal tumours is very unfortunate. " The term (cancer of the larynx) should be restricted to the so-called ' intrinsic ' group of cancers. The extrinsic group are of pharyngeal origin, require separate and distinct classification, and should be excluded from any discussion on laryngeal cancer" (Lederman, 1950).

*The Paterson-Kelly syndrome.*

Any comparison of cancer of the larynx in men and in women requires some consideration of the syndrome, confined to women, of dysphagia, due to " organic stenosis at the level of the cricopharyngeal sphincter or immediately beyond it " (Bingham and Logan, 1953), atrophy and ulceration of the mucous membranes of the

* Of 178 cases of intrinsic laryngeal cancer treated by teleradium at the Radiumhemmet, Stockholm, during 1935–45, 88·2 per cent were in men (Jacobsson, 1952).

mouth, tongue, and pharynx, angular stomatitis, hypochromic anaemia, achlorhydria, splenomegaly, and spoon nails, in which carcinoma of the post-cricoid region may develop in later years. This condition was first described independently by Paterson from Cardiff (1919) and by Brown Kelly from the Western Infirmary, Glasgow (1919) and has become known by the names of two later investigators as the "Plummer-Vinson" syndrome. Neither Paterson (1919, 1937) nor Brown Kelly (1919, 1931) suggest, either in their earlier or later papers, that this affection is especially common in their own districts, though any such frequency would have assisted recognition. This syndrome is of quite especial interest in cancer research because the features present during the earlier years may indicate a precancerous metabolic state, which may be treated successfully by improved feeding and administration of iron (See for instance Hallén, 1938 ; Waldenstrom, 1938, and Elkeles, 1942). The following section represents an attempt to collect the literature on the Paterson-Kelly syndrome in relation to cancer ; no attempt has been made to introduce any uniformity into the anatomical classification adopted by the various authors.

Ahlbom (1937) records, from the Radiumhemmet, Stockholm (1931–1936), the incidence of carcinoma of the post-cricoid region. (*a*) Cancers of the hypopharynx are more frequent in women, in whom also a much higher proportion (90 per cent) of these tumours affect the post-cricoid area, than in men (10 per cent) (Table IX). (*b*) Seventy-five per cent of cancers of the larynx and oesophagus in women are accompanied by other signs of the syndrome (Table X).

In a later paper Ahlbom (1941) defines hypopharyngeal cancers as those arising between the vallecula and the upper end of the oesophagus, excepting the

TABLE IX.—*Carcinoma of Meso- and Hypopharynx, Larynx and Oesophagus. Cases at the Radiumhemmet,* 1931–1936 (Ahlbom, 1937).

| Carcinoma. | Male. | Female. | |
|---|---|---|---|
| Tonsil | 16 | 7 | |
| Other mesopharynx | 0 | 8 | About 10 per cent of male and 90 per cent of female were post-cricoid. |
| Hypopharynx | 50 | 74 | |
| Larynx | 47 | 8 | |
| Oesophagus | 24 | 16 | |
| | 137 | 113 | |

TABLE X.—*Female Patients at the Radiumhemmet,* 1931–1936 (*Ahlbom,* 1937).

| Carcinoma. | Total cases. | Not examined for anaemia. | Examined. | Plummer-Vinson syndrome. | Simple achylic anaemia.* | Negative. |
|---|---|---|---|---|---|---|
| Tonsil | 7 | 2 | 5 | 3 | 0 | 2 |
| Other naso-pharynx | 8 | 3 | 5 | 5 | 0 | 0 |
| Hypopharynx | 74 | 25 | 49 | 37 | 5 | 7 |
| Larynx | 8 | 0 | 8 | 1? | 1? | 6 |
| Oesophagus | 16 | 10 | 6 | 3 | 0 | 3 |
| | 113 | 40 | 73 | 49 | 6 | 18 |

(Plummer-Vinson syndrome + Simple achylic anaemia: 55 = 75 per cent of those examined.)

* One must infer from the totals that these cases showed no other signs of the syndrome.

intrinsic laryngeal carcinomas. His series of 235 cancers of this region was derived from 129 women and 106 men seen at the Radiumhemmet during 1937–39, (Table XI). Sideropenia (this term includes the Plummer-Vinson syndrome and simple achlorhydric anaemia) . . . " in this country is to be regarded as the cancer-predisposing factor of decisive significance in these female patients. In practically

TABLE XI.—*Cancers of Hypopharynx, Radiumhemmet,* 1931–1947 (*Ahlbom,* 1941 *and Jacobsson* 1951).

| | | | | Cancers of hypopharynx. | | |
|---|---|---|---|---|---|---|
| | Male | Female | Female per cent of total. | Upper. | Lower (post-cricoid). | Sideropenia. |
| Ahlbom, 1931–1936 . | 50 | 74 | 60 | | 20 per cent of male<br>90 per cent of female | 90 per cent of post-cricoid. |
| „ 1931–1939 . | 106 | 129 | 55 | | "Practically all" hypopharyngeal cancers in sideropenic female | |
| Jacobsson, 1939–1947 | 119 | 203 | 63 | 85 per cent male | 20 per cent female | 10 per cent of male.<br>90 per cent of female. |

all of these women the site of the cancer is what is referred to as post-cricoid. This form is extremely rare in the male patients where the cancer develops in the sinus piriformis, the aryepiglottic folds, on the posterior arytenoid regions, as well as on and around the epiglottis." These two papers by Ahlbom (1937, 1941) contain valuable summaries of the literature.

Jacobsson (1951) has given more data from the Radiumhemmet (Table XI). He says: " The border line between the upper, and lower, hypopharynx, . . . " (as defined above by Ahlbom) " has been drawn at the superior border of the cricoid cartilage." The material consists of 322 cases (119 in males and 203, or 63 per cent, in females) ; about three-quarters of the tumours were in the lower hypopharynx, but to define the exact site of origin may not be possible. 85 per cent of the cases in the upper, and only 20 per cent of cases in the lower, hypopharynx were in males. " In our cases 90 per cent of the women and 10 per cent of the men showed definite signs and symptoms of sideropenia " (apparently this statement applies to tumours of all parts of the hypopharynx).

Jacobsson gives some figures from other countries. In Ireland, Norway, Scotland and Sweden, women provide 40 to 60 per cent of cancers of the hypopharynx, whereas in France and Switzerland this conditions in women is very rare. The range given is so wide (from 1 per cent in France to 60 per cent in Sweden) that one would like to be sure that the diagnostic criteria are the same. Hallén (1938) speaks of "the widespread achlorhydria in the northern provinces of Sweden."

Simpson (1939), whose cases " were all from Hull and the immediate area surrounding in the East Riding of Yorkshire " (personal communication), says: " on examining the records of the cases of this syndrome referred to me for dysphagia and oesophagoscopy, I find that at least 50 per cent either had or developed a carcinoma. Of the 18 cases seen, 10 had either a carcinoma or developed one while under observation. Of the 10 carcinomas, four were in the post-cricoid area, one was in the lower third of the oesophagus, and five were located at the cardiac end of the stomach and oesophagus."

Pilcher (1948) gives the following details of 100 consecutive cases of carcinoma of the pharynx seen at University College Hospital, London :

TABLE XII.—*Carcinoma of Pharynx* (Pilcher, 1948).

| | Epilaryngeal. | Post-cricoid. | Total. |
|---|---|---|---|
| Male | 70 { Pyriform fossa, 42; Lateral wall, 9 } | 0 | 70 |
| Female | 1 | 29 | 30 |

Lederman (1953) has discussed the anatomy of the pharyngo-laryngeal groove and sinus pyriformis. " Tumours arising from the third or lowest part of the groove can scarcely be distinguished from other tumours of the post-cricoid space and should be regarded as forming part of this latter group. . . . Tumours of the upper and mid parts of the groove can be conveniently regarded as a single group and the term " sinus pyriformis " cancer applied to this joint group, the " sinus pyriformis " for this purpose being regarded as extending from the upper border of the pharyngo-epiglottic fold above to the crico-pharyngeal fold below." He gives the following data, which show the sexual distribution, in his material, immediately above the post-cricoid area. The sex-ratio (15 : 1) is almost the same as that (13 : 1) in Stanford Cade's (1953) series (Table XIII).

Cancer of Sinus Pyriformis.
112 previously untreated cases.
Royal Cancer Hospital, London. 1933–1951.
Male 105. Female 7.

A full discussion of anatomical relationships has been given in a paper by Lederman (1954).

Stanford Cade (1953) gives the following analysis of 403 cases seen by him in London of squamous-celled carcinoma of the laryngo-pharynx.

TABLE XIII.—*Carcinoma of Laryngo-Pharynx,* 1930–1952 (*Stanford Cade,* 1953).

| | Total. | Men. | Women. |
|---|---|---|---|
| Epilaryngeal | 77 | 70 | 7 |
| Lateral pharyngeal | 47 | 42 | 5 |
| Pyriform fossa | 167 | 155 | 12 |
| Post-cricoid | 112 | 30 | 82 |
| Total | 403 | 297 | 106 |

Bingham and Logan (1953) in Belfast, met with 16 cases of the syndrome in 5 years, of which 2 developed post-cricoid carcinoma ; they say that the condition is " relatively common in the north of Ireland . . . the social class and the level of intelligence are below average . . . Courses of iron produce no permanent improvement . . . if not repeated twice a year . . . these people have not been able to eat meat for years . . . the lumen at the stricture being sometimes only a few millimetres in diameter."

*Diet in relation to the Paterson-Kelly syndrome.*

Stocks (1939) has examined dietetic conditions in North Wales. " The statistical association between cancer and the amount of fresh milk and certain fresh vegetables in dietaries which was found some years ago by comparing the diets of 400 cancer patients with those of 400 control patients of the same ages from the same localities (Stocks and Karn, 1933) may help to provide a key to this. Fresh milk and vegetables seem to be very poorly represented in the diet of the bulk of the population of North Wales."

Visitors to Wales, who make no claim to have investigated the matter upon a statistical basis, may get the impression that white bread-and-butter and tea predominates in the diet of many women. Hallén (1938) speaking of diet in relation to the Paterson-Kelly syndrome says: " . . . the problem may be of great importance for the cure of the widespread achlorhydria in the northern provinces of Sweden. According to recent calculations large fractions of the population live on a diet very poor in iron (men 17·5 mg. iron pro die, women only 11 mg)." On a possible anti-carcinogenic action of milk see Hoch-Ligeti (1946, 1952).

Ahlbom (1937) says: " Im Material des Radiumhemmets ist das Verhältnis zwischen der Anzahl von Männern und Frauen bei den in Rede stehenden Karzinomgruppen* ungefähr 1 : 1 bei den ärmeren Patienten, aber ungefähr 5 : 1 bei den Privatpatienten. Unter den ungefähr 100 Fällen von Plummer-Vinsonschen Syndrom und einfacher Achylie-Anämïe mit Karzinom, die wir seit dem Jahre 1931 diagnostizierten, waren nur 4 Privatpatienten, eine bemerkenswert geringe Verhältniszahl. Was in der Diät der ärmeren schwedischen Bevölkerung fehlt, sind vor allem eisenhaltige Vegetabilien, Obst und Fleisch."

Bingham and Logan (1953) say " the incidence of hypochromic anaemia in Northern Ireland is considerable and is to be attributed to the inadequate nutrition common in middle-aged women in Northern Ireland and to uncorrected menorrhagia."

A valuable paper, based upon 300 cases of the Paterson-Kelly syndrome observed at the Radiumhemmet, with numerous references to the literature, has been published by Lindrall (1953).

### SUMMARY.

1. The mortality due to cancer of the larynx has shown little change in the last 20 years ; this contrasts with the well-known increase in that due to cancer of the lung.

2. Mortality due to cancer of the lung and larynx in the County Boroughs, Urban Districts and Rural Districts of different parts of England and Wales is analysed for the peiiod 1946–1949.

3. The Standard Mortality Ratio (S.M.R.) for cancer of the lung in both sexes, and of the larynx in males, increases with increasing urbanization, that is to say, is greater in the County Boroughs than the Urban Districts, and in the Urban Districts than the Rural Districts. Cancer of the female larynx shows exactly the reverse relationship.

4. These trends apply equally when the figures are analysed according to the separate regions, but there are differences between the regions, which may or may not be due to differences in degrees of urbanization undetected by the classification we have used. In particular the S.M.R.s for Wales are very low for the lung and male larynx but very high for the female larynx. The urbanization-gradient is less steep for the female lung than for the male lung.

* Lip, mouth, pharynx, larynx, oesophagus.

5. The mortality due to cancer of the male lung is positively correlated with the numbers of persons per acre in the Rural Districts of the 11 regions of England and Wales.

6. The significance of these findings is discussed in the light of the possible aetiology of cancer of the lung and larynx.

7. The anatomical and sexual distribution of 964 cases of cancer of the larynx is tabulated. Cancer of the larynx in men, and in women, are to a large extent different diseases, the so-called extrinsic group being more frequent in women.

8. The literature upon the relation of the Paterson-Kelly syndrome to cancer, and upon possible dietetic and social factors in this condition, is summarized.

We are greatly indebted to Dr. W. P. D. Logan and Mr. P. A. Phillips, of the General Register Office, for a large part of the material dealt with in this paper. We are very grateful to Dr. R. B. Terry for data from the records of this hospital and to Mr. V. E. Negus for those from his own practice. The investigation has been supported by generous grants from the British Empire Cancer Campaign and the Anna Fuller Fund.

### REFERENCES.

AHLBOM, H. E.—(1937) *Acta radiol.*, **18**, 163.—(1941) *Ibid.*, **22**, 155.
BINGHAM, J. A. W., AND LOGAN, J. S.—(1953) *Brit. med. J.*, ii, 650.
BROWN KELLY, A.—(1919) *J. Laryng.*, **34**, 285.—(1931) *Ibid.*, **46**, 326.
CADE, S.—(1953) *Proc. Roy. Soc. Med.*, **46**, 769.
ELKELES, A.—(1942) *Brit. J. Radiol.*, **15**, 1.
HALLÉN, L.—(1938) *Acta med. scand.*, Suppl., **90**, 398.
HOCH-LIGETI, C.—(1946) *Cancer Res.*, **6**, 563.—(1952) Texas Reports Biol. Med., **10**, 996.
JACOBSSON, F.—(1951) *Acta radiol.*, **35**, 1.—(1952) *Ibid.*, **38**, 143.
KENNAWAY, E. L., AND KENNAWAY, N. M.—(1951) *Brit. J. Cancer*, **5**, 153.
*Idem* AND WALLER, R. E.—(1953) *Acta Un. int. Cancr.*, **9**, 485.
LEDERMAN, M.—(1950) *Ibid.*, **6**, 1249.—(1953) *J. Laryng.*, **67**, 641.—(1954) *Ibid.*, **68**, 333.
LINDRALL, N. (1953) *Acta radiol.*, **39**, 17.
PATERSON, D. R.—(1919) *Ibid.*, **34**, 289.—(1937) *Ibid.*, **52**, 75.
PILCHER, R. S.—(1948) *Proc. Roy. Soc. Med.*, **41**, 445.
Registrar General's Decennial Supplement, England and Wales, 1931. Part II*a*, p. 37 (1938).
SEMON, F.—(1907) *Brit. med. J.*, i, 241.
SIMPSON, R. R.—(1939) *Proc. Roy. Soc. Med.*, **32**, 1447.
STOCKS, P.—(1939) *Ann. Rep. Brit. Emp. Cancer Campgn.*, **15**, 26.—(1952) *Brit. J. Cancer*, **6**, 99.
*Idem* AND KARN, M. N.—(1933) *Ann. Eugen., Camb.*, **5**, 237.
THOMSON, ST. C. AND COLLEDGE, L. (1930) 'Cancer of the Larynx.' London (Kegan Paul).
WALDENSTRÖM, J.—(1938) *Acta med. scand.*, Suppl., **90**, 380.